# Treatment outcomes of patients with chronic hepatitis C receiving sofosbuvir-based combination therapy within national hepatitis C elimination program in the country of Georgia

**DOI:** 10.1186/s12879-019-4741-5

**Published:** 2020-01-10

**Authors:** Tengiz Tsertsvadze, Amiran Gamkrelidze, Muazzam Nasrullah, Lali Sharvadze, Juliette Morgan, Shaun Shadaker, Lia Gvinjilia, Maia Butsashvili, David Metreveli, Vakhtang Kerashvili, Marina Ezugbaia, Nikoloz Chkhartishvili, Akaki Abutidze, Valeri Kvaratskhelia, Francisco Averhoff

**Affiliations:** 1 Infectious Diseases, AIDS and Clinical Immunology Research Center, Tbilisi, Georgia; 2 Ivane Javakhishvili Tbilisi State University, Faculty of Medicine, Tbilisi, Georgia; 3 National Center for Disease control and public health, 99, Kakheti highway, 0198 Tbilisi, Georgia; 4 Emerging Infections Program, Centers for Disease Control and Prevention (CDC), Division of Viral Hepatitis National Center for HIV, Hepatitis, STD&TB Prevention, Atlanta, USA; 5 Hepatology Clinic HEPA, Tbilisi, Georgia; 6 CDC Foundation, Georgia Hepatitis C Elimination Program, Tbilisi, Georgia; 7 Clinic NeoLab, Tbilisi, Georgia; 8 Medical Center Mrcheveli, Tbilisi, Georgia; 9 Ministry of Labor, Health and Social Affairs of Georgia, Tbilisi, Georgia

**Keywords:** HCV, Elimination, DAAs, SVR, Georgia

## Abstract

**Background:**

Georgia has one of the highest HCV prevalence in the world and launched the world’s first national HCV elimination programs in 2015. Georgia set the ambitious target of diagnosing 90% of people living with HCV, treating 95% of those diagnosed and curing 95% of treated patients by 2020. We report outcomes of Sofosbuvir (SOF) based treatment regimens in patients with chronic HCV infection in Georgia.

**Methods:**

Patients with cirrhosis, advanced liver fibrosis and severe extrahepatic manifestations were enrolled in the treatment program. Initial treatment consisted of SOF plus ribavirin (RBV) with or without pegylated interferon (INF). Sustained virologic response (SVR) was defined as undetectable HCV RNA at least 12 weeks after the end of treatment. SVR were calculated using both per-protocol and modified intent-to-treat (mITT) analysis. Results for patients who completed treatment through 31 October 2018 were analyzed.

**Results:**

Of the 7342 patients who initiated treatment with SOF-based regimens, 5079 patients were tested for SVR. Total SVR rate was 82.1% in per-protocol analysis and 74.5% in mITT analysis. The lowest response rate was observed among genotype 1 patients (69.5%), intermediate response rate was achieved in genotype 2 patients (81.4%), while the highest response rate was among genotype 3 patients (91.8%). Overall, SOF/RBV regimens achieved lower response rates than IFN/SOF/RBV regimen (72.1% vs 91.3%, *P* < 0.0001).

In multivariate analysis being infected with HCV genotype 2 (RR =1.10, CI [1.05–1.15]) and genotype 3 (RR = 1.14, CI [1.11–1.18]) were associated with higher SVR. Patients with cirrhosis (RR = 0.95, CI [0.93–0.98]), receiving treatment regimens of SOF/RBV 12 weeks, SOF/RBV 20 weeks, SOF/RBV 24 weeks and SOF/RBV 48 weeks (RR = 0.85, CI [0.81–0.91]; RR = 0.86, CI [0.82–0.92]; RR = 0.88, CI [0.85–0.91] and RR = 0.92, CI [0.87–0.98], respectively) were less likely to achieve SVR.

**Conclusions:**

Georgia’s real world experience resulted in high overall response rates given that most patients had severe liver damage. Our results provide clear evidence that SOF plus IFN and RBV for 12 weeks can be considered a treatment option for eligible patients with all three HCV genotypes. With introduction of next generation DAAs, significantly improved response rates are expected, paving the way for Georgia to achieve HCV elimination goals.

## Background

Globally, an estimated 71 million people are chronically infected with hepatitis C virus (HCV), and 400,000 die annually from hepatitis C-related liver diseases [1]. Management of HCV infection has been revolutionized after the availability of direct acting antivirals (DAAs), and Sofosbuvir (SOF) was the first widely introduced DAA [2, 3]. Clinical trials have demonstrated high efficacy of SOF-based regimens in patients infected with genotypes 1–6 [4–8].

Georgia has one of the highest HCV prevalence rates among general population in the world [9], and launched the world’s first national HCV elimination program in 2015 [10]. The elimination program has adopted a comprehensive strategy that addresses both prevention and treatment of HCV infection. A key component of the program is the provision of DAAs free of charge to all Georgian citizens; this was made possible through an agreement with Gilead Sciences to donate DAAs. Georgia has set itself the ambitious target of diagnosing 90% (135,000 persons) of people living with HCV, treating 95% (128,000 persons) of those diagnosed and curing 95% (121000) of treated patients by 2020 [9]. We report outcomes of SOF-based treatment regimens in patients with chronic HCV infection in the country of Georgia.

## Methods

All Georgians aged 18 years or older that are infected with HCV are eligible for the free of charge treatment program. The hepatitis C elimination program was launched on 28 April 2015. All patients treated from launch through 31 October 2018 are included in the analysis. Treatment-naive and experienced patients with cirrhosis (including decompensated cirrhosis), advanced liver fibrosis, severe extrahepatic manifestations, HCV re-infection after liver transplantation and HIV-coinfection were prioritized for enrollment in the treatment program. Initially, DAA treatment was exclusively SOF based and included ribavirin (RBV) with or without pegylated interferon, depending on the HCV genotype, per national guidelines. From February 2016, more effective, interferon free DAA combination - sofosbuvir and ledipasvir (SOF/LDV) was introduced, and treatment regimens were revised. Beginning in June 2016, treatment criteria were relaxed allowing enrollment of all HCV infected persons regardless of level of liver fibrosis, to be treated. Treatment guidelines were established by a committee composed of treatment experts from Georgia in consultation with international experts. Based on eligibility of interferon therapy all HCV genotype 1 and 3 patients received SOF plus, Pegylated interferon (IFN) and RBV for 12 weeks or SOF plus RBV for 24 weeks. HCV genotype 2 treatment naïve patients without cirrhosis were treated with the 12-week combination of SOF plus RBV, while cirrhotic patients and those with prior treatment failure received the 12-week regimen of SOF plus IFN and RBV or the 20-week regimen of SOF plus RBV based on eligibility of interferon. Patients with decompensated cirrhosis received SOF plus RBV for 48 weeks.

Treatment was initially limited to four sites in Tbilisi, and later expanded with sites from other cities within Georgia; by October 2018, 31 sites were providing HCV treatment in the country. The HCV treatment program providers also participated in Project ECHO (Extension for Community Healthcare Outcomes).

A national HCV treatment database was established, which collected standard data for each patient enrolled in treatment program. Each treatment site was responsible for data entry for each enrolled patient. Data were de-identified and sociodemographic, clinical and laboratory data were extracted from national HCV treatment database. Characteristics measured included: age, gender, HCV RNA, FIB-4 test score, METAVIR score, HBsAg, treatment regimen, HCV genotype and city where treatment was provided. Sustained virologic response (SVR) was defined as undetectable HCV RNA at least 12 weeks after the end of treatment. The presence of cirrhosis was confirmed by vibration-controlled transient elastography or acoustic radiation force impulse elastography (ARFI) compatible with stage F4 fibrosis (≥14.5 kpa)_by METAVIR. Decompensated cirrhosis was defined as the presence of current or past ascites, hepatic encephalopathy and variceal haemorrhage etc. SVRs were calculated using both per-protocol and modified intent-to-treat (mITT) analysis. Per-protocol approach included only those with complete SVR data, while in mITT analysis persons discontinuing treatment were also included. Persons who died or had no SVR test > 24 weeks after completing treatment were excluded from analysis.

### Statistical analysis

All analyses were performed with SAS version 9.3 software (SAS Institute, Inc., Cary, NC, USA). Variables were categorized as follows: age category: 18–44, 45–60, and > 60; HCV RNA category: < 800,000 IU/mL vs. ≥800,000 IU/mL; FIB-4 test: <1.45, 1.45–3.25 and > 3.25; METAVIR score: <F4 and F4. We used the chi-square or Fisher’s exact to compare differences in categorical variables with SVR. We performed a multivariate logistic-regression analysis involving baseline demographic, clinical and laboratory characteristics to identify independent predictors of SVR. A *p*-value < 0.05 was considered significant. The final model included variables associated (*p* < 0.05) with SVR in the bivariate analysis. The results are presented with a Risk ratio (RR) and 95% Confidence intervals (CIs). Results for patients who completed treatment and tested for SVR through 31 October 2018 were analyzed. The study was approved by the Institutional review board of the Infectious Diseases, AIDS and Clinical Immunology Research Center, Tbilisi.

## Results

A total of 7342 patients with chronic HCV infection received SOF-based therapy from April 28, 2015 until October 31, 2018 and 5079 had complete SVR data.

The pretreatment demographics, clinical and laboratory characteristics of patients with complete SVR data are described in Table 1. Most patients, 2838 (55.9%) were age 45–60 years, 4381 (86.3%) were males and 2783 (57.9%) had stage F4 fibrosis (by METAVIR). Overall, 1724 (33.9%) of the patients had HCV genotype 1, followed by HCV genotype 3, 2305 (45.4%) and HCV genotype 2, 1047 (20.6%). Only 3 patients were infected with HCV genotype 4. Majority of patients were treated with IFN/SOF/RBV for 12 weeks (52.1%), followed by SOF/RBV for 24 weeks (27.9%), SOF/RBV for 20 weeks (7.8%), SOF/RBV for 12 weeks (7.2%), and SOF/RBV for 48 weeks (5.0%).

**Table 1:** Baseline characteristics of adult persons with complete SVR data treated with SOF-based regimens by HCV genotypes within the national hepatitis C elimination program, April 28, 2015 – October 31, 2018

Characteristic	TOTAL		Genotype 1		Genotype 2		Genotype 3		Genotype 4	
	n	%	n	%	n	%	n	%	n	%
Age category, n (%)										
18–45	1635	32.2	386	22.4	299	28.6	948	41.1	2	66.7
45–60	2838	55.9	944	54.8	630	60.2	1264	54.8	.	.
60+	606	11.9	394	22.9	118	11.3	93	4.0	1	33.3
Gender, n (%)										
Female	698	13.7	486	28.2	101	9.6	110	4.8	1	33.3
Male	4381	86.3	1238	71.8	946	90.4	2195	95.2	2	66.7
HCV RNA categories, n (%)										
< 800,000 IU/mL	2922	57.7	901	52.5	625	59.8	1393	60.5	3	100.0
≥ 800,000 IU/mL	2145	42.3	816	47.5	420	40.2	909	39.5	.	.
FIB-4 Test										
< 1.45	200	5.7	65	6.0	51	7.0	84	5.0	.	.
1.45–3.25	1763	50.2	491	45.0	403	55.1	868	51.5	1	50.0
> 3.25	1546	44.1	535	49.0	277	37.9	733	43.5	1	50.0
Metavir score										
< F4	2021	42.1	676	39.8	516	50.9	827	39.6	2	66.7
F4	2783	57.9	1021	60.2	497	49.1	1264	60.4	1	33.3
Liver function tests, n (%)										
ALT >2 X ULN	2585	51.0	731	42.5	466	44.6	1385	60.2	3	100.0
AST >2 X ULN	2604	51.4	783	45.6	442	42.3	1376	59.8	3	100.0
Billirubin >1.1 mg/dL	4423	87.3	1520	88.5	928	88.8	1972	85.7	3	100.0
Albumin < 35 g/L	2001	39.5	670	39.0	469	44.9	862	37.4	.	.
INR >1.49	687	13.6	260	15.1	132	12.6	295	12.8	.	.
Co-infections, n (%)										
HBsAg+	108	2.2	28	1.7	19	1.9	61	2.8	.	.
HBsAg-	4777	97.8	1666	98.3	985	98.1	2123	97.2	3	100.0
Treatment regimen, n (%)										
IFN/SOF/RBV (12 wk)	2646	52.1	905	52.5	240	22.9	1500	65.1	1	33.3
SOF/RBV (12 wk)	364	7.2	3	0.2	360	34.4	1	0	.	.
SOF/RBV (20 wk)	395	7.8	3	0.2	392	37.4	.	.	.	.
SOF/RBV (24 wk)	1418	27.9	695	40.3	7	0.7	714	31	2	66.7
SOF/RBV (48 wk)	256	5	118	6.8	48	4.6	90	3.9	.	.
City of treatment site, n (%)										
Tbilisi	3800	74.8	1294	75.1	819	78.2	1684	73.1	3	100
Kutaisi	362	7.1	148	8.6	72	6.9	142	6.2	.	.
Batumi	501	9.9	177	10.3	67	6.4	257	11.1	.	.
Zugdidi	328	6.5	90	5.2	81	7.7	157	6.8	.	.
Gori	42	0.8	6	0.3	5	0.5	31	1.3	.	.
Rustavi	40	0.8	9	0.5	3	0.3	28	1.2	.	.
Lanchkhuti	4	0.1	.	.	.	.	4	0.2	.	.
Gurjaani	2	0	.	.	.	.	2	0.1	.	.

*SOF* Sofosbuvir, *RBV* Ribavirin, *IFN* Interferon

A total of 521 persons discontinued treatment, with the most common causes for not completing treatment being death (48.8%; *n* = 254), self-discontinuation (19.6%; *n* = 102), and loss to follow up (15.9%; *n* = 83). Among those who died during treatment, the majority 299/521 (57.4%) had severe liver disease (METAVIR scores of F3 or F4).

A total of 5079 persons with complete SVR data and 521 persons who discontinued treatment, were included in treatment efficacy analysis (total 5600 persons). Total SVR rate was 82.1% (4170/5079) in per-protocol analysis and 74.5% (4170/5600) in mITT analysis.

Of those with an SVR12, the lowest response rate was observed among genotype 1 patients (1198/1724; 69.5%), intermediate response rate was achieved in genotype 2 patients (852/1047; 81.4%), while the highest response rate was among genotype 3 patients (2117/2305; 91.8%). There were only 3 patients with genotype 4 and all were cured.

**Fig. 1:**
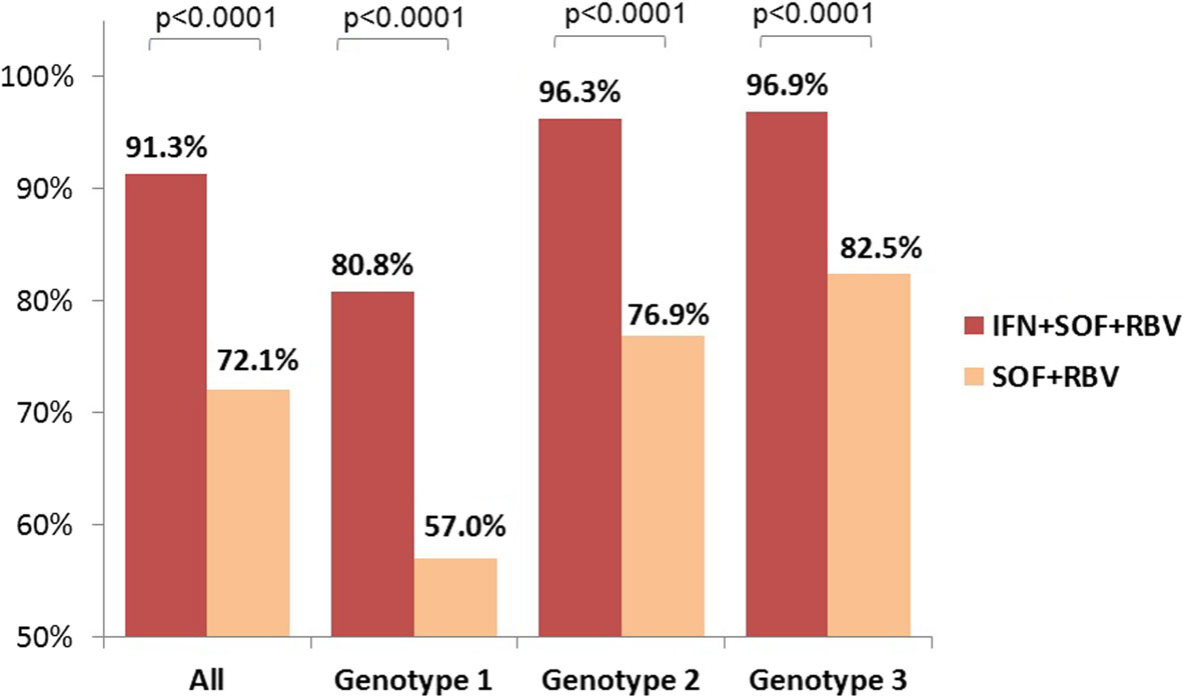
SVR rates by treatment regimens and genotype (*n* = 5076)

Overall, SOF/RBV regimens achieved lower response rates than IFN/SOF/RBV regimen (72.1% vs 91.3%, *P* < 0.0001). This difference was seen in all genotypes (57.0% vs 80.8%, *P* < 0.0001 for genotype 1; 76.9% vs 96.3%, *P* < 0.0001 for genotype 2 and 82.5% vs 96.9%, *P* < 0.0001 for genotype 3 respectively) (Fig. 1).

Multivariate analysis (Table 2) showed that when controlling those factors which were significantly associated with SVR in bivariate analysis, being infected with HCV genotype 2 (RR =1.10, CI [1.05–1.15], *P* = 0.001) and genotype 3 (RR = 1.14, CI [1.11–1.18], *P* < 0.0001) were associated with higher SVR. Patients with cirrhosis (RR = 0.95, CI [0.93–0.98], *P* < 0.0001), receiving treatment regimens of SOF/RBV 12 weeks, SOF/RBV 20 weeks, SOF/RBV 24 weeks and SOF/RBV 48 weeks (RR = 0.85, CI [0.81–0.91], *P* < 0.0001; RR = 0.86, CI [0.82–0.92], *P* < 0.0001; RR = 0.88, CI [0.85–0.91], *P* < 0.0001 and RR = 0.92, CI [0.87–0.98], *P* = 0.005, respectively) were less likely to achieve SVR.

**Table 2:** Treatment outcomes and associated factors among adult persons with complete SVR data receiving SOF-based regimens within the national hepatitis C elimination program, April 28, 2015 – October 31, 2018

	Total N	Achieved SVR		Bivariate analysis			Multivariate analysis		
		N	%	RR	95% CI	*p* value	RR	95% CI	*p* value
Age category									
18–45	1635	1440	88.07	1					
46–60	2838	2259	79.60	0.90	0.88–0.93	<0.0001			
60+	606	471	77.72	0.88	0.84–0.92	<0.0001			
Gender									
Female	698	560	80.23	1					
Male	4381	3610	82.40	1.03	0.99–1.07	0.18			
HCV Genotype									
1	1724	1198	69.49	1			1		
2	1047	852	81.38	1.17	1.12–1.22	<0.0001	1.10	1.05–1.15	<0.0001
3	2305	2117	91.84	1.32	1.28–1.37	<0.0001	1.14	1.11–1.18	<0.0001
4	3	3	100.00	–	–	–	–	–	–
HCV RNA categories, n (%)									
< 800,000 IU/mL	2922	2408	82.41	1					
≥ 800,000 IU/mL	2145	1754	81.77	0.99	0.97–1.02	0.56			
FIB-4 Tests									
< 1.45	200	177	88.50	1			1		
1.45–3.25	1763	1573	89.22	1.01	0.96–1.06	0.76	1.00	0.93–1.07	0.95
> 3.25	1546	1166	75.42	0.85	0.80–0.90	<0.0001	0.95	0.87–1.02	0.17
Metavir score									
< F4	2021	1761	87.14	1			1		
F4	2783	2161	77.65	0.89	0.87–0.97	<0.0001	0.95	0.93–0.98	0.0001
Co-infections									
HBsAg-	4777	3897	81.58	1					
HBsAg+	108	89	82.41	1.01	0.92–1.10	0.82			
Treatment regimen									
IFN/SOF/RBV (12 wk)	2646	2416	91.31	1			1		
SOF/RBV (12 wk)	364	276	75.82	0.83	0.78–0.88	<0.0001	0.85	0.81–0.91	<0.0001
SOF/RBV (20 wk)	395	302	76.46	0.84	0.79–0.89	<0.0001	0.86	0.82–0.92	<0.0001
SOF/RBV (24 wk)	1418	979	69.04	0.76	0.73–0.78	<0.0001	0.88	0.85–0.91	<0.0001
SOF/RBV (48 wk)	256	197	76.95	0.84	0.79–0.90	<0.0001	0.92	0.87–0.98	0.005
City of treatment site									
Tbilisi	3800	3127	82.29	1			1		
Kutaisi	362	272	75.14	0.91	0.86–0.97	0.004	0.96	0.92–1.01	0.10
Batumi	501	435	86.83	1.06	1.02–1.10	0.005	1.01	0.97–1.05	0.60
Zugdidi	328	258	78.66	0.96	0.90–1.01	0.13	0.96	0.92–1.00	0.07
Gori	42	40	95.24	1.16	1.08–1.24	<0.0001	1.01	0.87–1.17	0.91
Rustavi	40	32	80.00	0.97	0.83–1.14	0.72	0.95	0.80–1.13	0.55
Lanchkhuti	4	4	100.00	–	–	–	–	–	–
Gurjaani	2	2	100.00	–	–	–	–	–	–

*SOF* Sofosbuvir, *RBV* Ribavirin, *IFN* Interferon, *CI* Confidence interval, *RR* Risk ratio, *SVR* Sustained virologic response

## Discussion

This study from Georgia is one of the largest real-world cohorts examining outcomes of HCV treatment with SOF based regimens, among patients with severe liver disease. We assessed real-world efficacy of SOF plus RBV with or without IFN in these difficult-to-treat patients with chronic hepatitis C. Our study demonstrated that SOF-based regimens can result in high overall SVR rates, similar to SVR rates achieved in clinical trials [11, 12]. While newer combination DAAs are now available, SOF is now one of the most readily available DAAs worldwide, at affordable prices in many low middle income countries, and as such, these findings have relevance today. In particular, the acceptable SOF plus RBV outcomes among the most severely ill patients, regardless of genotype are highly relevant.

In our study response rates among patients with HCV genotype 2 were lower than reported in clinical trials and real-life studies which showed high efficacy of SOF plus RBV combination treatment among HCV genotype 2 patients including those with cirrhosis and/or treatment experience [8, 12–15]. Lower efficacy of treatment in genotype 2 patients may have been associated with a reported high prevalence of HCV recombinant form 2 k/1b among Georgian HCV genotype 2 patients [16]; these patients do not respond well to standard treatment for genotype 2 and regimens used for genotype 1 seem to be more effective [17]. Therefore there is a need for reassessing existing modalities for the management of HCV genotype 2 infection, especially in areas with high prevalence of HCV recombinant form 2 k/1b [18].

We observed high cure rates in HCV genotype 3 patients that are one of the most challenging subpopulations to treat [19]. IFN-based regimens were superior to SOF/RBV alone. The results of clinical trials showed that HCV genotype 3 patients achieved higher SVR12 rates with a 12 week SOF and RBV in combination with IFN that patients who were treated with SOF and RBV alone [12].

Our findings support use of a 12 week regimen of SOF plus RBV in combination with IFN as a treatment option for eligible HCV genotype 3 patients in settings, where new highly potent and well-tolerated DAAs against genotypes 2 and 3 are not available. Our results suggest the use of SOF/RBV combination for 24 weeks as an option for patients who cannot tolerate IFN.

After examining host and viral factors we found that presence of cirrhosis, and receiving IFN-free regimens were associated with lower SVR in a multivariable model. The low rates of response among cirrhotic patients is consistent with previous studies.

One strength of this study is the large number of patients as well as standardized treatment guidelines and standardized data collection. The diversity of our cohort with respect to sex, age, and genotype distribution makes our findings generalizable, reflecting reported real-world outcomes. Our study has several limitations. First, data from patients in whom prior treatment had failed, was not collected. Second, liver fibrosis was assessed by multiple noninvasive indices, each of which have limitations on accuracy [20–22]. The national treatment database, which captures information on all hepatitis C patients enrolled in the program, provides accurate treatment related information on a national level. However it does not contain detailed information on some variables, including comorbidities (diabetes mellitus, kidney failure, extrahepatic manifestations etc.) as well as nature of deaths, adverse events and reasons of self-discontinuation. Also data available in the national system has limited ability to answer questions as to why people are lost to follow-up along the continuum of care. Significant number of patients who were lost to follow-up after treatment completion is a serious challenge of the treatment program. However, in 2017 the program offered SVR assessment free of charge that would lead to reducing missing SVR data.. Despite notable progress of the Georgia HCV elimination program, challenges to Georgia achieving the national targets for HCV elimination by 2020 remain. Pangenotypic DAAs that are effective across the different genotypes of HCV introduced in late 2018 could have a substantial impact on improving access and simplifying diagnosis and treatment.

## Conclusion

In conclusion, in this large cohort study, a combination of SOF and weight-based RBV with or without IFN appeared to be an effective regimen to treat chronic HCV-infected patients, especially for HCV Genotype 2 and 3 patients. SOF formed the foundation of the HCV elimination program in Georgia. Cure rates in patients without cirrhosis were high, which are comparable with those reported in clinical trials. However, consistent with previous studies, the presence of liver cirrhosis were associated with lower SVR12 rates. Our results provide clear evidence that SOF plus IFN and RBV for 12 weeks can be considered a treatment option for eligible patients with all three HCV genotypes. With the introduction of next generation DAAs, replacement of IFN-based regimens by IFN-free regimens and significantly improved response rates are expected, paving the way for Georgia to achieve the goal of HCV elimination. High cure rates obtained with SOF/LDV combinations for all HCV genotypes within Georgia program highlights effectiveness of service delivery model, which is based on simplified modalities that can be successfully replicated in non-specialty settings, which is important in light of ongoing decentralization process. Strong governmental commitment coupled with effective local and international partnerships provide a basis for turning the ambitious goal of elimination into reality.

## Data Availability

The data that support the findings of this study are property of Georgia’s HCV elimination program. In case the data is requested, please contact scientific committee of Georgia’s HCV elimination program (Secretary Dr. Tinatin Kuchuloria, email: drkuchuloria@yahoo.com).

## Abbreviations

CI: Confidence intervals
DAAs: Direct acting antivirals
HCV: Hepatitis C virus
INF: Pegylated interferon
mITT: modified intent-to-treat
RBV: Ribavirin
RR: Risk ratio
SOF: Sofosbuvir
SVR: Sustained virologic response
